# Recurrent Erythema Multiforme Induced by the Combination of Pembrolizumab and Radiation

**DOI:** 10.7759/cureus.19202

**Published:** 2021-11-02

**Authors:** Austin B Ambur, Jason Mammino, Rajiv Nathoo

**Affiliations:** 1 Dermatology, Kansas City University-Graduate Medical Education Consortium/Advanced Dermatology and Cosmetic Surgery, Oviedo, USA

**Keywords:** mycoplasma pnemoniae, herpes simplex virus type 1, clinical dermatology, general dermatology, non small cell lung cancer, radiotherapy (rt), erythema multiforme

## Abstract

Erythema multiforme is a self-limited condition that is characterized by the rapid onset of symmetric targetoid lesions. This hypersensitivity reaction is commonly triggered by herpes simplex virus and *Mycoplasma pneumoniae* infections. Pembrolizumab is an anti-programmed cell death protein 1 (PD-1) inhibitor that is associated with drug-induced erythema multiforme. Radiotherapy is a rarely reported trigger of erythema multiforme. We report a case of recurrent pembrolizumab and radiotherapy-induced erythema multiforme in a patient with stage IV non-small cell lung cancer. This study aimed to highlight a unique cause of erythema multiforme and to review the common radiation-induced cutaneous reactions.

## Introduction

Erythema multiforme (EM) is a hypersensitivity reaction that is characterized by a rapid onset of symmetric targetoid lesions. The most common causes are herpes simplex virus (HSV) and *Mycoplasma pneumoniae* infections. Additional triggers include drugs and physical sources [1]. Radiotherapy is one physical source that has rarely been reported to trigger EM [2-4]. Pembrolizumab is an anti-programmed cell death protein 1 (PD-1) inhibitor that results in cutaneous adverse reactions in up to one-third of patients. These cutaneous reactions include a range of dermatologic manifestations including erythema multiforme, bullous pemphigoid, lichenoid dermatitis, spongiotic dermatitis, vitiligo-like lesions, acneiform eruptions, and lupus-like reaction [5]. The disease is typically self-limited, however, relapses may occur. We report a case of recurrent combined pembrolizumab and radiotherapy-induced EM in a patient with stage IV non-small cell lung cancer.

## Case presentation

**Figure 1:**
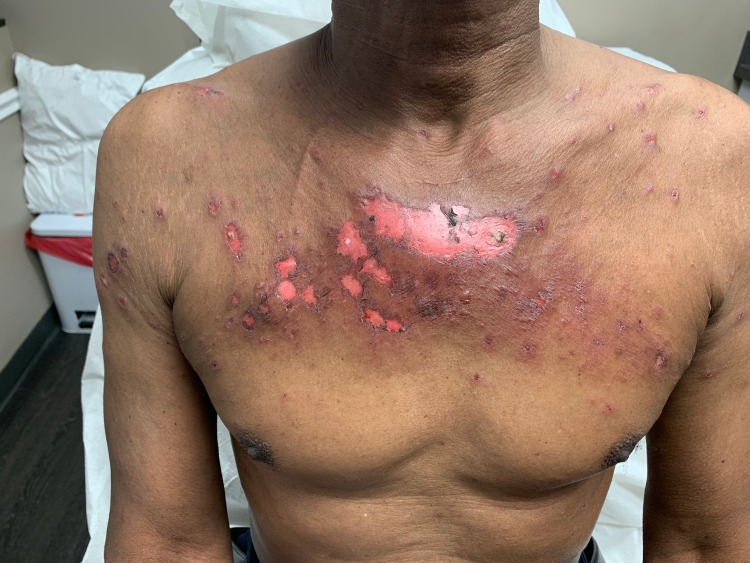
Eroded plaques to the mid-chest with surrounding targetoid and vesicular lesions.

**Figure 2:**
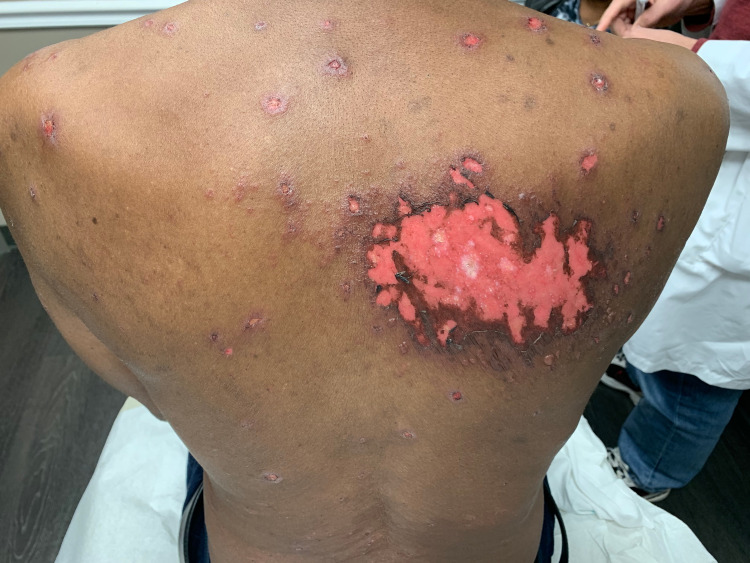
Eroded plaques to the upper back with surrounding targetoid and vesicular lesions.

A 68-year-old black male with a history of stage IV non-small cell lung cancer with metastasis to his spine and kidney status post partial lobectomy three years prior, benign prostate hyperplasia, and hypertension presented with recurrent EM minor. Seven months prior to his presentation, oncology initiated a regimen of cisplatin, pemetrexed, and pembrolizumab. After six cycles of this regimen, treatment was discontinued due to intolerable side effects including vitiliginous and eczematous cutaneous changes. He was therefore treated with five days of stereotactic body radiotherapy (SBRT) to his chest. He received a total dose of 5500 cGy. The patient initially presented to our clinic five weeks post-radiation. He had an expanding eroded vesicular eruption concentrated to the site of irradiation of two weeks duration (Figures 1, 2).

**Figure 3:**
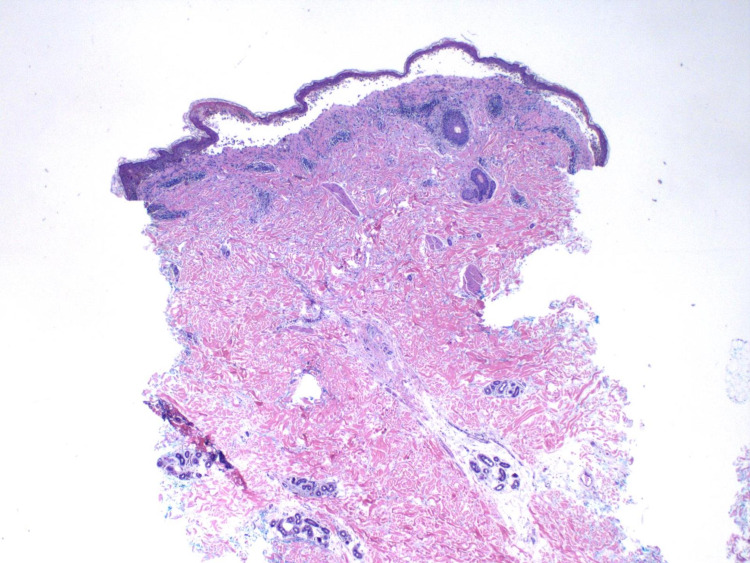
Hematoxylin and eosin-stained tissue section of skin from upper back (original magnification x2). The section of skin shows showing scattered necrotic keratinocytes within the epidermis with necrosis of the epidermis, separation of the dermo-epidermal junction, and an underlying interface and superficial perivascular inflammatory cell infiltrate of lymphocytes and rare eosinophils.

**Figure 4:**
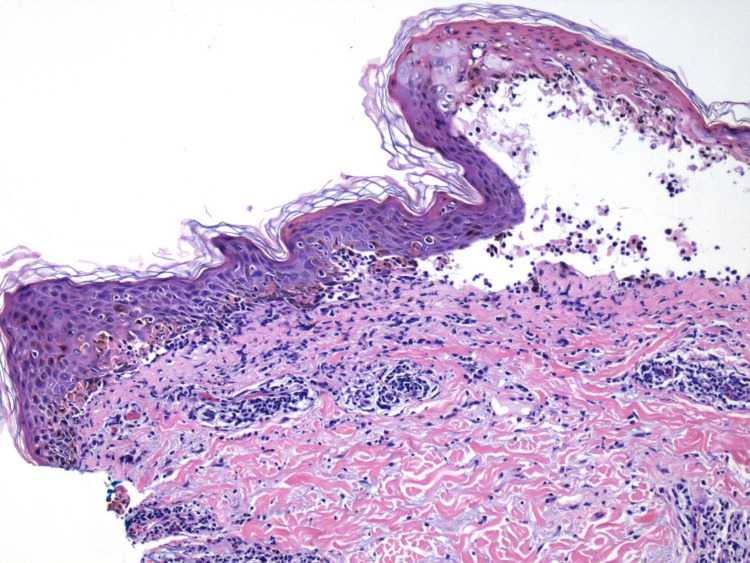
Hematoxylin and eosin-stained tissue section of skin from upper back (original magnification x10). The section of skin shows scattered necrotic keratinocytes within the epidermis with necrosis of the epidermis, separation of the dermo-epidermal junction, and an underlying interface and superficial perivascular inflammatory cell infiltrate of lymphocytes and rare eosinophils.

Hematoxylin-eosin (H&E) staining via punch biopsy performed on a lesional vesicle to the upper back showed scattered necrotic keratinocytes within the epidermis with necrosis of the epidermis, separation of the dermo-epidermal junction, and an underlying interface and superficial perivascular inflammatory cell infiltrate of lymphocytes and rare eosinophils consistent with EM (Figures 3, 4).

**Figure 5:**
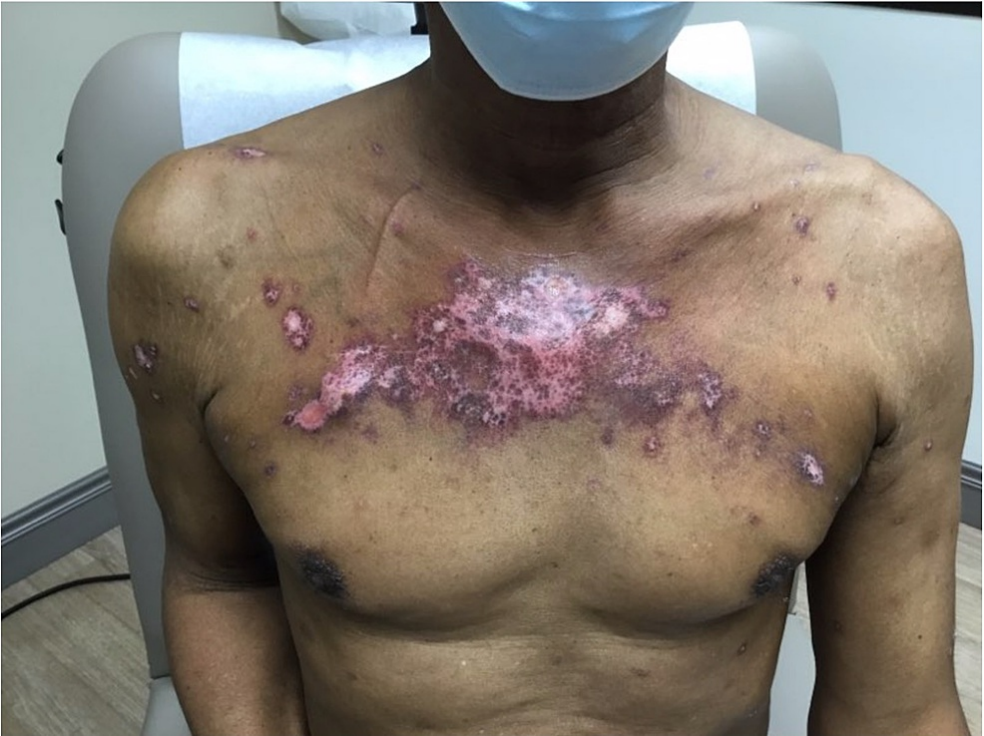
Healing central wounds with surrounding areas of post-inflammatory pigmentary changes located on the mid-chest.

**Figure 6:**
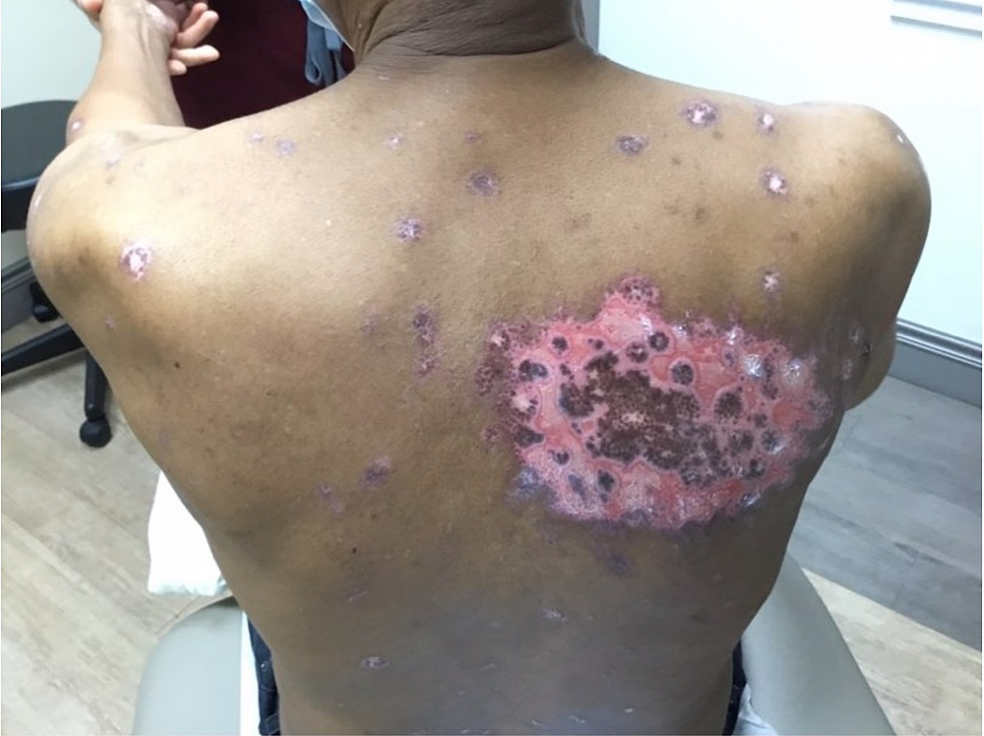
Healing central wounds with surrounding areas of post-inflammatory pigmentary changes on the upper back.

Direct immunofluorescence (DIF) was negative for other vesiculobullous diseases and bacterial culture was negative. Viral studies for herpes-simplex-virus (HSV) and *Mycoplasma pneumoniae* were remarkable for elevations in IgG without IgM. Daily medications included alfuzosin but were otherwise unremarkable for any new triggers. Pembrolizumab-induced EM has been reported to occur between three to 35 months after starting treatment and was postulated to be the initiating trigger of EM [5]. Radiotherapy was also implicated in the development of EM as the skin lesions started in the irradiated area. Our patient was treated with topical 0.1% triamcinolone ointment BID and showed significant improvement (Figures 5, 6).

**Figure 7:**
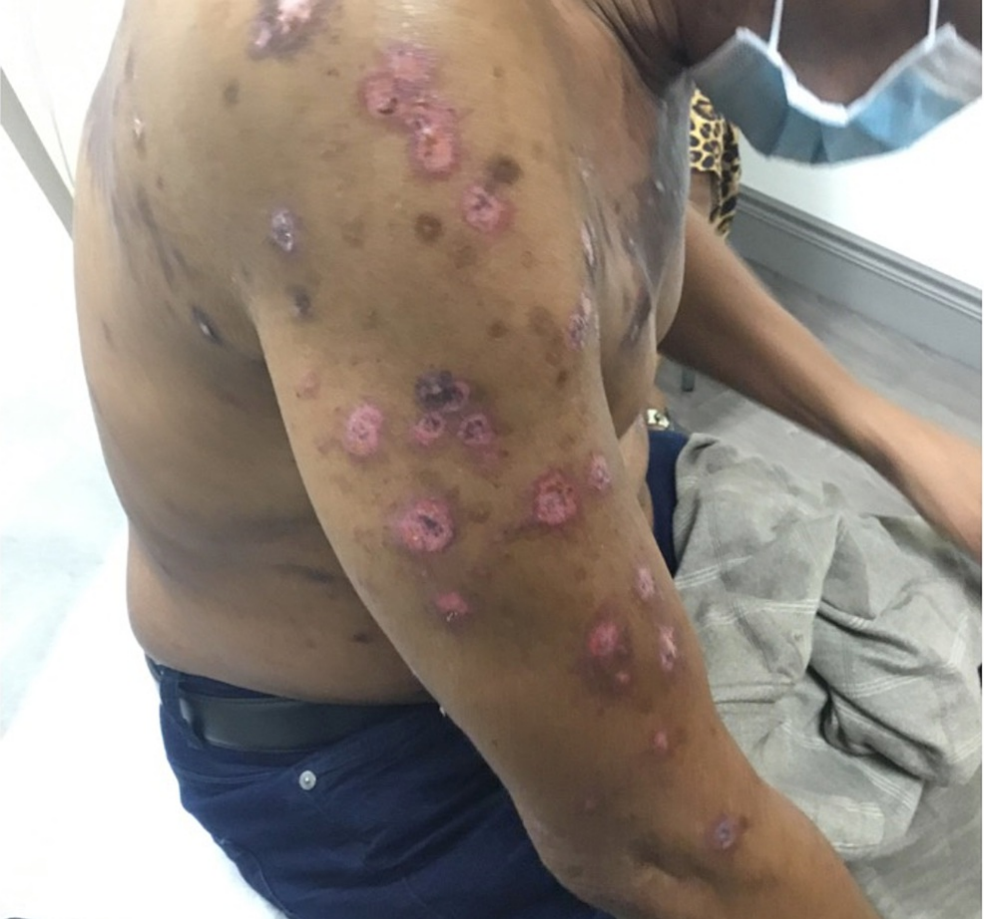
Numerous eroded papular targetoid lesions on the right arm.

At five weeks after the initial visit, the patient presented with multiple subtle erythematous papules at sites distant from the main truncal lesions. Despite increasing the potency of his topical steroid to betamethasone dipropionate, the lesions evolved quickly into papular targetoid lesions covering much of the upper body, sparing the head (Figure 7).

Given the recurrent nature of the lesions, he was started on daily valacyclovir 500 mg twice daily. Our patient continued to develop recurrent lesions despite suppressive antiviral therapy and therefore was ultimately started on dapsone.

## Discussion

EM is characterized by the rapid onset of numerous symmetric fixed red papules that evolve into targetoid lesions. EM is subdivided into EM minor and EM major. They are distinguished by the absence of mucosal involvement and systemic symptoms in EM minor. The disease is self-limited and typically resolves within two weeks for EM minor and six weeks for EM major [1]. It is important to note that EM is characterized by papules as it may be mistaken for Stevens-Johnson syndrome or toxic epidermal necrolysis, which are characterized by macular lesions with a higher likelihood of mucosal involvement. The most common cause of EM minor is HSV-1 whereas EM major is more commonly associated with *Mycoplasma pneumoniae* infection [1]. Several drugs have been described in the literature in association with EM, including non-steroidal anti-inflammatory drugs (NSAIDs), sulfonamides, anticonvulsants, and allopurinol [1]. Pembrolizumab is a PD-1 inhibitor that is a less commonly reported trigger of EM [5]. The PD-1 receptor is expressed on the active cluster of differentiation 4 (CD4^+^) T cells and plays an important role in down-regulating T cell proliferation, reducing cytotoxic activity, and the expansion of regulatory T cells [6]. PD-1 inhibition may also enhance the expression of Fas receptors on keratinocytes [7]. Interaction of Fas ligand on T cells with Fas receptors on keratinocytes leads to apoptosis of keratinocytes and may be involved in the pathogenesis of pembrolizumab-induced EM. Physical sources that may induce EM include trauma, cold, and UV and X-ray irradiations [1]. The cause of radiotherapy-induced EM is unknown. It has been postulated that radiotherapy may induce an immune-based hypersensitivity reaction by the toxic breakdown products during treatment [3]. Radiotherapy may also lead to a hypersensitivity reaction by preferentially impairing T-suppressor cells. The hypersensitivity reaction induced by radiotherapy may have had synergistic and/or complementary contributions to the immune dysregulation induced by pembrolizumab and the development of EM. Several conditions known to be associated with radiation include radiodermatitis, recall phenomenon, Stevens-Johnson syndrome, and toxic epidermal necrolysis [8]. It is important to recognize the etiology, unique phenotypical findings, and timing of the cutaneous reaction associated with various radiation-induced cutaneous reactions (Table 1).

**Table 1:** Radiation-induced cutaneous reactions

Inflammatory Condition	Latency Period	Affected Region	Clinical Morphology
Erythema multiforme	10-20 days	Irradiated field + dissemination	Maculopapular
Radiodermatitis	2 weeks	Irradiated field	Erythema/desquamation/ulceration
Recall phenomenon	1 week to months	Irradiated field	Erythema/desquamation/edema
Stevens-Johnson syndrome/toxic epidermal necrolysis	10-20 days	Irradiated field + dissemination	Maculopapular + mucositis

Initial treatment includes topical steroids in mild cases and systemic steroids in severe diseases. Chronic suppressive antiviral therapy can be implemented for recurrent disease. Azathioprine, thalidomide, dapsone, cyclosporine, mycophenolate mofetil, and psoralen and ultraviolet light A (PUVA) may be used if the condition is resistant to antiviral therapy [1].

## Conclusions

We present a case of recurrent combined pembrolizumab and radiotherapy-induced EM minor to highlight a unique cause of EM that has been rarely described. We hypothesize that pembrolizumab was the initiating trigger for EM and was exacerbated by radiotherapy. We also use this case to demonstrate the possible recalcitrant nature of the disease. It is important to understand the various radiation-induced cutaneous reactions and their distinct clinical features to make the correct diagnosis and reduce the delay in appropriate therapy.
